# The transverse aortic constriction heart failure animal model: a systematic review and meta-analysis

**DOI:** 10.1007/s10741-020-09960-w

**Published:** 2020-04-25

**Authors:** Lena Bosch, Judith J. de Haan, Marissa Bastemeijer, Jennifer van der Burg, Erik van der Worp, Marian Wesseling, Margarida Viola, Clémene Odille, Hamid el Azzouzi, Gerard Pasterkamp, Joost P.G. Sluijter, Kimberley E. Wever, Saskia C.A. de Jager

**Affiliations:** 1 Laboratory of Experimental Cardiology, University Medical Center Utrecht, Heidelberglaan 100, 3584 CX Utrecht, Netherlands; 2 Central Diagnostics Laboratory, University Medical Center Utrecht, Utrecht, The Netherlands; 3 UMC Utrecht Regenerative Medicine Center, Circulatory Health Laboratory, University Utrecht, Utrecht, Netherlands; 4 SYstematic Review Centre for Laboratory animal Experimentation (SYRCLE), Department for Health Evidence, Nijmegen Institute for Health Sciences, Radboud University Medical Center, Nijmegen, The Netherlands; 5 Laboratory of Translational Immunology, Department of Immunology, University Medical Center Utrecht, Utrecht, The Netherlands; 6 Department of Molecular Genetics, Erasmus University Medical Center, Rotterdam, Netherlands

**Keywords:** Transverse aortic constriction, Hear failure, Animal model, Systematic review, Meta-analysis

## Abstract

The transverse aortic constriction (TAC) model is frequently used to study adverse cardiac remodeling upon pressure overload. We set out to define the most important characteristics that define the degree of cardiac remodeling in this model. A systematic review and meta-analyses were performed on studies using the TAC mouse/rat model and reporting echocardiographic outcome parameters. We included all animal studies in which a constriction around the transverse aorta and at least one of the predefined echocardiography or MRI outcome parameters were assessed. A total of 502 articles and > 3000 wild-type, untreated animals undergoing TAC were included in this study and referenced to a control group. The duration of aortic constriction correlated to the degree of adverse remodeling. However, the mouse data is strongly biased by the preferential use of male C57Bl/6 mice (66% of studies). Furthermore, mostly ketamine/xylazine anesthetics, 27G needle constriction, and silk sutures are used. Nonetheless, despite the homogeneity in experimental design, the model contained a substantial degree of heterogeneity in the functional outcome measures. When looking at study quality, only 12% reported randomization, 23% mentioned any sort of blinding, 25% adequately addressed the outcomes, and an amazingly low percentage (2%) showed sample size calculation. Meta-analyses did not detect specific study characteristics that explained the heterogeneity in the reported outcome measures, however this might be related to the strong bias towards the use of specific mouse lines, sex as well as age or to poor reporting of characteristics of study quality.

**Electronic supplementary material:**

The online version of this article (10.1007/s10741-020-09960-w) contains supplementary material, which is available to authorized users.

## Introduction

Approximately 23 million people worldwide suffer from heart failure (HF), and this incidence will further increase the coming decades. [1] The development of HF is characterized by a process of adverse cardiac remodeling. [2] To study this process, several animal models are available and a commonly used model is a pressure overload–induced HF by transverse aortic constriction (TAC). First described by Rockman et al in 1991, the TAC model was designed to study the mechanisms behind cardiac hypertrophy. [3] Besides hypertrophy, the model is characterized by cardiac fibrosis, a limited amount of inflammation, and eventually cardiac dilation and HF. [4] Animals subjected to TAC exhibit variable severity of adverse cardiac remodeling [5], and multiple study characteristics can potentially influence the response to TAC. Variability exists in selected animal species, genetic background, sex, follow-up time, and severity of constriction. To which extend these variables affect the degree of cardiac remodeling is not known; however, they are important for interpretation of published data and may aid to design optimization of the TAC model. Since the introduction of murine echocardiography, numerous studies using the TAC model were published reporting cardiac dimensions and function as outcome parameters. [6]

Translational failure is common in HF research; therapies with efficacy in preclinical models have failed when applied to human clinical trials. Multiple factors are involved in these disappointing outcomes, including limitations in preclinical animal models, hindering the development of new therapies for HF. [7] Therefore, in this systematic review and meta-analyses, we sought to evaluate the influence of animal characteristics and methodological differences of TAC surgery on echocardiographic outcome parameters. A systematic review of animal studies using the TAC model may provide insight into a number of issues currently impeding translation, such as the characteristics of the animal model and the quality of the published studies. Previously, systematic reviews of preclinical studies have proven useful in design optimization of both preclinical and clinical studies. [8] In this systematic review and meta-analysis, we set out to analyze the influence of variables such as strain, sex, and surgery characteristics on adverse cardiac remodeling after TAC.

## Methods

### Search strategy

A detailed protocol was published online (PROSPERO registration number CRD42017079553; https://www.crd.york.ac.uk/prospero/display_record.php?RecordID=79553) before the start of this systematic review and provided as supplement to this manuscript. [9] This systematic review is based on published results of animal studies that used a transverse aortic constriction (TAC) model in untreated, wild-type animals to induce adverse cardiac remodeling.

### Amendments to the review protocol

Data extraction was performed in duplicate by two individual researchers for 57 (11.4%) randomly selected included studies, after which the percentage deviation between reviewers was calculated. Since this percentage was very low, 0.8% for numerical data and 1.2% for data extracted using digital ruler software, data extraction for the remaining 443 studies was performed by a single reviewer. We chose to leave the secondary outcome analysis because of the results of the primary outcome analysis. Furthermore, we only took mouse for our primary analysis because of limited number of articles for other species and for the understandability of the mean difference instead of standardized mean difference.

### Search and study selection

Pubmed and EMBASE were searched for transverse aortic constriction and its synonyms on 12-04-2017 (see supplemental table 1&2 for all search terms). A second search was done on 20-03-2019, to update this review. The animal filters developed by SYRCLE were used to select only animal studies. [10] Duplicates were removed in Endnote. We included all animal studies in which TAC surgery was performed, with a constriction around the transverse aorta between the brachiocephalic artery and left common carotid artery, and at least one of the following outcome parameters assessed by echocardiography or MRI were included: end diastolic volume (EDV) or end diastolic diameter (EDD), end systolic volume (ESV) or end systolic diameter (ESD), and/or ejection fraction (EF) or fractional shortening (FS). All animal species and sexes were included. Only studies with a relevant control group (sham surgery, baseline measurements or untreated control animals) were included. Only full publications with original data were included. All studies without TAC or the functional outcome parameters mentioned above were excluded. Animals with comorbidities, genetically modified animals, animals undergoing co-intervention such as compound or solvent administration were excluded. Abdominal, ascending, and descending aortic constriction, angiotensin II infusion, and other ways of inducing hypertension/pressure overload were excluded. Conference abstracts, letters to the editor, and editorials were excluded. Articles not reporting the number of animals were excluded. The systematic review platform SyRF (syrf.org.uk) was used for study selection and extraction of data. Articles were first screened for eligibility based on their title and abstract, and eligible articles were then screened for final inclusion based on full text. Title/abstract screening and full-text screening were performed by at least two independent researchers, and discrepancies were additionally assessed by a third investigator.

### Data collection

From each publication, the following data was extracted: species, genetic background, sex, age, weight, number of animals, TAC duration, characteristics of TAC procedure namely suture material, constriction diameter, anesthetic, follow-up time, and whether a minimally invasive surgery technique was used defined as entering the thorax through the 2nd intercostal space. When articles reported a range of, for instance, age or weight, the average was taken. Outcome data were extracted for the primary outcomes EDD, EDV, ESD, and ESV and the secondary outcomes FS and EF by echocardiography or MRI, cardiac hypertrophy, and survival. For each outcome, group averages, standard deviation (SD), and number of animals per group (*n*) were extracted. If the standard error was reported, it was converted to SD. When multiple time points were assessed, only data from the latest time point was extracted. When data was presented only graphically, a digital ruler was used for extraction (Universal Desktop Ruler; AVPSOFT).

### Risk of bias assessment

To assess risk of bias, SYRCE’s Risk of Bias tool was used [11] with some adaptations. We included reporting of sample size calculations as an additional study quality indicator. In addition, we extracted data on the reporting of any measure of randomization, blinding, or conflict of interest statement. Furthermore, we assessed if outcome data were adequately addressed, defined by reporting the number of animals included in the analysis separately for each outcome measure.

### Data synthesis and statistical analysis

Data were analyzed using STATA/SE, (version 11; StataCorp, College Station, TX, USA). For EDD, EDV ND ESD, and ESV, we calculated the effect size as mean difference (MD) between the TAC group and non-TAC control group (consisting of a mixture of nontreated and sham controls) with corresponding 95% confidence interval (MD [95%CI]). To account for anticipated heterogeneity, effect sizes were pooled using random effects meta-analysis, taking into account the precision of individual studies and the variation between studies and weighing each study accordingly. Heterogeneity was quantified using *I*^2^ and *R*^2^ statistics. Pre-specified subgroup analyses were performed using meta-regression if at least two subgroups contained ≥ 5 comparisons. If meta-regression indicated that a subgroup variable explained a significant proportion of between-study heterogeneity, differences between individual strata were determined by performing a *t* test on the MD or RR and 95% CI of these strata.

## Results

### Study selection process

**Fig. 1:**
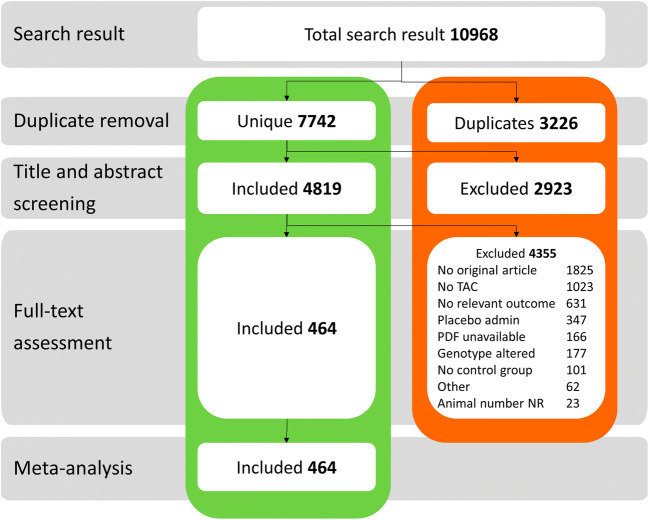
Flow chart of study selection process. Our systematic search in Pubmed and EMBASE yielded 7742 unique publications. After title and abstract screening, articles were screened full text of which 4355 were excluded based on exclusion criteria. Data from 464 articles was included in meta-analysis and quality assessment. TAC transverse aortic constriction, admin administration, other possible reasons: additional surgery such as ovariectomy, methods not mentioned clearly, for instance duration of TAC not stated, NR not reported

After removal of duplicates, a total of 7742 papers were included, after which 3226 articles were excluded (flow chart in Fig. 1) based on title and abstract. During full-text screening, 3351 articles were further excluded, see Fig. 1 for the number of articles excluded per exclusion criterion. A total of 468 articles were used for data extraction.

### Animal characteristics

**Fig. 2:**
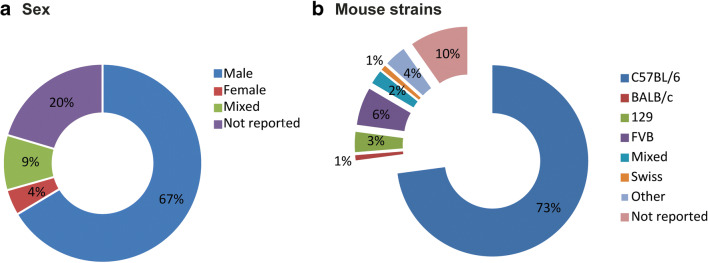
Distribution of sex and mouse strain. Distribution of **a** sex of the animals in all included studies and **b** mouse strains of included mouse studies (absolute numbers of a total of 500)

The 464 included articles contained a total of 500 comparisons of an experimental group exposed to TAC versus a group measured at baseline or undergoing sham surgery. All included experiments were performed in rodents, of which 95% (472/500 comparisons) in mice and 5% (28/500) in rats. Data were predominantly obtained from male animals (66%, 332/500). Females were used in 5% (21/500) of comparisons, 8% (45/500) used a mixed group of animals, and 21% (102/500) did not report the sex of the animals used (Fig. 2a). Various different mouse strains were used (Fig. 2b). Most commonly C57BL/6 mice (67%, 344/472), for 10% (46/472) of the comparisons, the mouse strain was not specified. Median age of the mice was 10 weeks, ranging from 3 to 56 weeks, and 7 weeks for rats (range: 3–12 weeks). The median body weight was 25 g for mice (range: 20–36 g) and 155 g for rats (range: 45–250 g).

### Surgery characteristics

**Fig. 3:**
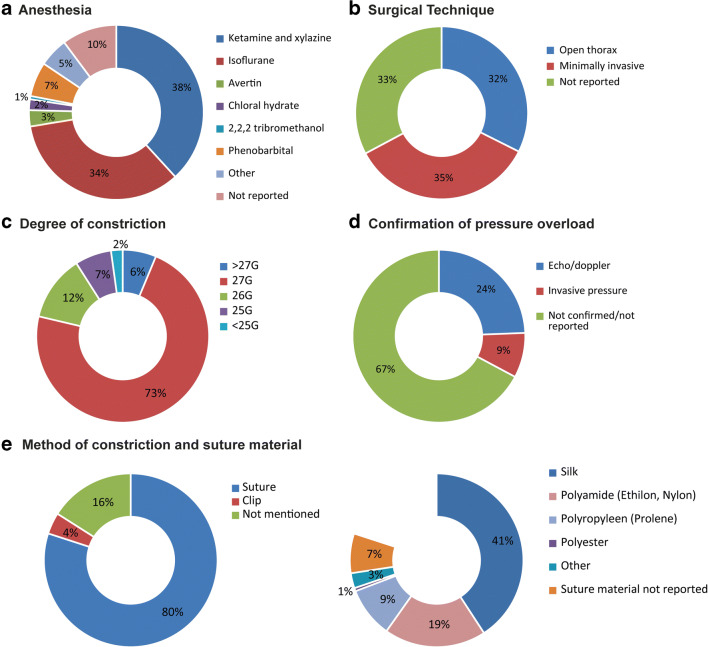
Study characteristics. Distribution of **a** anesthetics used during TAC or sham surgery, **b** surgical technique, **c** degree of constriction, **d** confirmation of pressure overload, and **e** method of constriction and suture materials (absolute numbers of a total of 500)

As the vast majority of studies was performed in mice and in order to improve clarity and readability of the manuscript, we decided to continue further on sub analysis in mouse studies only. For TAC or sham surgery, mainly ketamine and xylazine (≈40%, 191/500) or isoflurane (≈30%, 171/500) were used as anesthetic (Fig. 3a). Thirty-five percent of the experiments were performed using minimally invasive techniques, 32% was not performed minimally invasively, and 33% did not specify this (respectively 174, 162, and 164 out of 500 comparisons). The most frequently used technique to apply TAC was placing a suture around the transverse aorta (80%, 400/500). Of the studies reporting suture type, 53% (204/383 comparisons) used a silk suture (Fig. 3b), while 25% (95/383) made use of polyamide and 12% (47/383) of a prolene suture. In 4% (20/500) of the comparisons, a clip was used to apply TAC, and 16% (80/500) did not mention the material or method used for TAC induction. The TAC diameter was reported in 373/500 comparisons in gauge (G) of the needle used, which ranged from 17 to 30G. In most cases, a 27G needle was used (71%; 265/373), followed by 26G (12%; 45/373) and 25G (7%; 26/373). In 33% of cases (154/500), confirmation of TAC after surgery was reported, which was performed by echo/doppler in 122 of these comparisons and in 42 by invasive pressure measurements. In 66% (336/500) of the studies, either confirmation of pressure overload on the heart was not assessed or it was not reported (Fig. 3c). After surgery, follow-up time ranged from 3 to 280 days. Most experiments used a follow-up time of 28 days (21%; 107/500), followed by 14 and 56 days (both 10%; 53/500) and 7 days (6%; 32/500). For functional outcome assessment, most comparisons (96%; 387/500) used 2D echocardiography. In a small proportion of the studies (3%, 13/500 comparisons), MRI was used.

### Study quality assessment

**Fig. 4:**
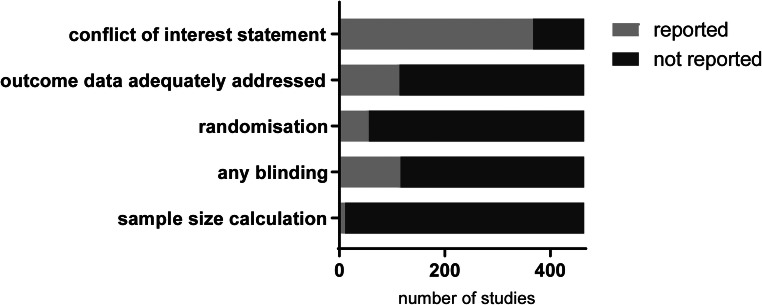
Data quality. Reporting of study quality indicators; conflict of interest statement, outcome data adequately addressed, blinding, randomization, and sample size calculation in the 464 included articles

The overall reporting of study quality indicators is presented in Fig. 4. Out of the total of 464 included studies, 342 (74%) included a conflict of interest statement, of which the vast majority (325, 95%) stated to have no conflict of interest. Only 23% (*n* = 108) studies reported the number of animals included in the analysis separately for each outcome measure (depicted as outcome data adequately addressed). Therefore, it cannot be ruled out that the majority of studies have excluded a number of animals for some of the outcome assessments. Unfortunately, the (predefined) criteria for exclusion were not reported. A limited number of studies reported randomization of animals (12%) or blinding (23%) of the investigators during the study at any level (allocation of the animals, blinding of researchers performing the experiment, or blinding during follow-up analysis). Only 11 studies (2%) reported a sample size calculation. Taken together, these data implicate a substantial risk of bias in the majority of published articles using the TAC model.

### Meta-analysis

**Fig. 5:**
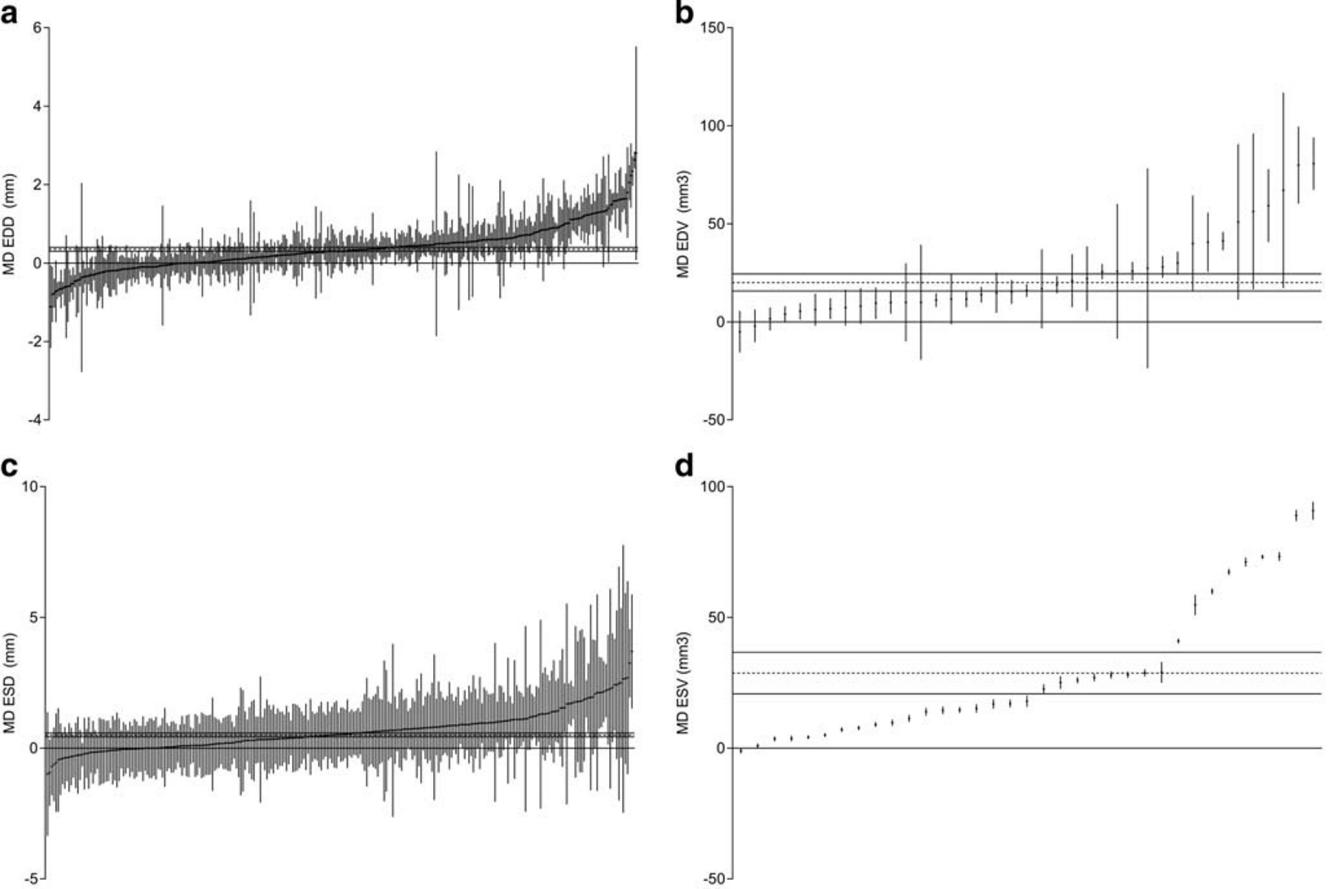
TAC induces severe structural remodeling of the heart. Forest plots of the effect of TAC on **a** end diastolic diameter (EDD; *n* = 314 comparisons), **b** end diastolic volume (EDV; *n* = 39), **c** end systolic diameter (ESD; *n* = 270), and **d** end systolic volume (ESV; *n* = 35). Data are presented as raw mean difference (MD) and 95% confidence intervals (95%CI). In all analyses, the pooled MD and 95%CI (horizontal dotted line and black line) indicate an increase outcome after TAC, when compared with controls

Despite the substantial risk of bias and the preference for using male C57Bl/6 mice, we investigate what the differences are in response to pressure overload between the sexes and different mouse strains. With these data in hand, we aim to aid to validated decision making regarding the choice for a specific animal model, strain, and sex. TAC induces severe structural remodeling of the heart, and we defined end diastolic (end diastolic diameter (EDD) and volume (EDV)) and end systolic diameter (ESD) and volume (ESV)) function as primary outcome measures. In a total of 341 comparisons with 3370 mice subjected to TAC, we observed an increase in EDD of 0.35 mm [0.3–0.4; *I*^2^ 91.2% *p* < 0.0001, Fig. 5a] compared with 2984 control mice. Total 398 mice subjected to TAC displayed an average EDV increase of 20.09 μl [15.7–24.4; *I*^2^ 92.8% *p* < 0.0001, Fig. 5b] compared with 356 control mice from a total of 39 studies/comparisons. When assessing systolic function, ESD increased with 0.50 mm [0.43–0.58; *I*^2^ 16.0% *p* = 0.017, Fig. 5c] in 2615 mice subjected to TAC referenced to 2383 control mice derived from 270 studies. Based on 35 studies ESV was on average increased by 28.74 μl [20.8–36.68; *I*^2^ 99.9% *p* < 0.0001, Fig. 5d] based on 345 mice subjected to TAC compared with 299 control mice.

### Subgroup analysis

Subgroup analyses were performed to identify sources of between-study heterogeneity. Table 1 summarizes the influence of the following study characteristics on the level of heterogeneity: sex, strain, and confirmation of the TAC. None of the investigated variables explained a significant proportion of the heterogeneity present in the overall analysis. Due to low number of comparisons for ESV/EDV, we did not investigated heterogeneity in these outcome parameters.

**Table 1:** Influence of: sex, strain, and confirmation of the TAC on the level of heterogeneity in EDD and ESD

	EDD			ESD		
	#	MD (mm)	[95%CI]	#	MD (mm)	[95%CI]
Sex	*p* = 0.41, adj. *R*^2^ = − 0.44%			*p* = 0.85 adj. *R*^2^ = − 3.38%		
Female	13	0.26	− 0.02, 0.55	8	0.58	0.13, 1.04
Male	217	0.38	0.31, 0.45	179	0.53	0.43, 0.62
Mixed	36	0.35	0.18, 0.53	24	0.48	0.24, 0.73
Not reported	75	0.27	0.15, 0.39	59	0.45	0.29, 0.61
Mouse strain	*p* = 0.04, adj. *R*^2^ = 2.84%			*p* = 0.11 adj. *R*^2^ = 5.51%		
129	15	0.14	− 0.12, 0.39	14	0.45	0.29, 0.61
Balb/c	2	− 0.23	− 1.05, 0.60	3	0.53	0.43, 0.62
C57BL/6	250	0.40	0.34, 0.47	194	0.53	0.43, 0.62
FVB	22	0.11	− 0.11, 0.32	15	0.58	0.13, 1.04
Mixed	7	0.10	− 0.33, 0.52	6	0.53	0.43, 0.62
Other	12	0.21	− 0.08, 0.50	12	0.53	0.43, 0.62
Swiss	3	− 0.03	− 0.61, 0.55	3	0.45	0.29, 0.61
Not reported	30	0.21	0.20, 0.58	23	0.45	0.29, 0.61
TAC confirmed	*p* = 0.63, adj. *R*^2^ = − 0.36%			*p* = 0.66 adj. *R*^2^ = − 1.52%		
Echo/doppler	85	0.34	0.22, 0.45	63	0.51	0.35, 0.68
Invasive pressure	33	0.27	0.10, 0.45	29	0.40	0.17, 0.64
Not reported	223	0.37	0.30, 0.43	178	0.52	0.42, 0.62

### Influence of sex and strain on TAC model

**Fig. 6:**
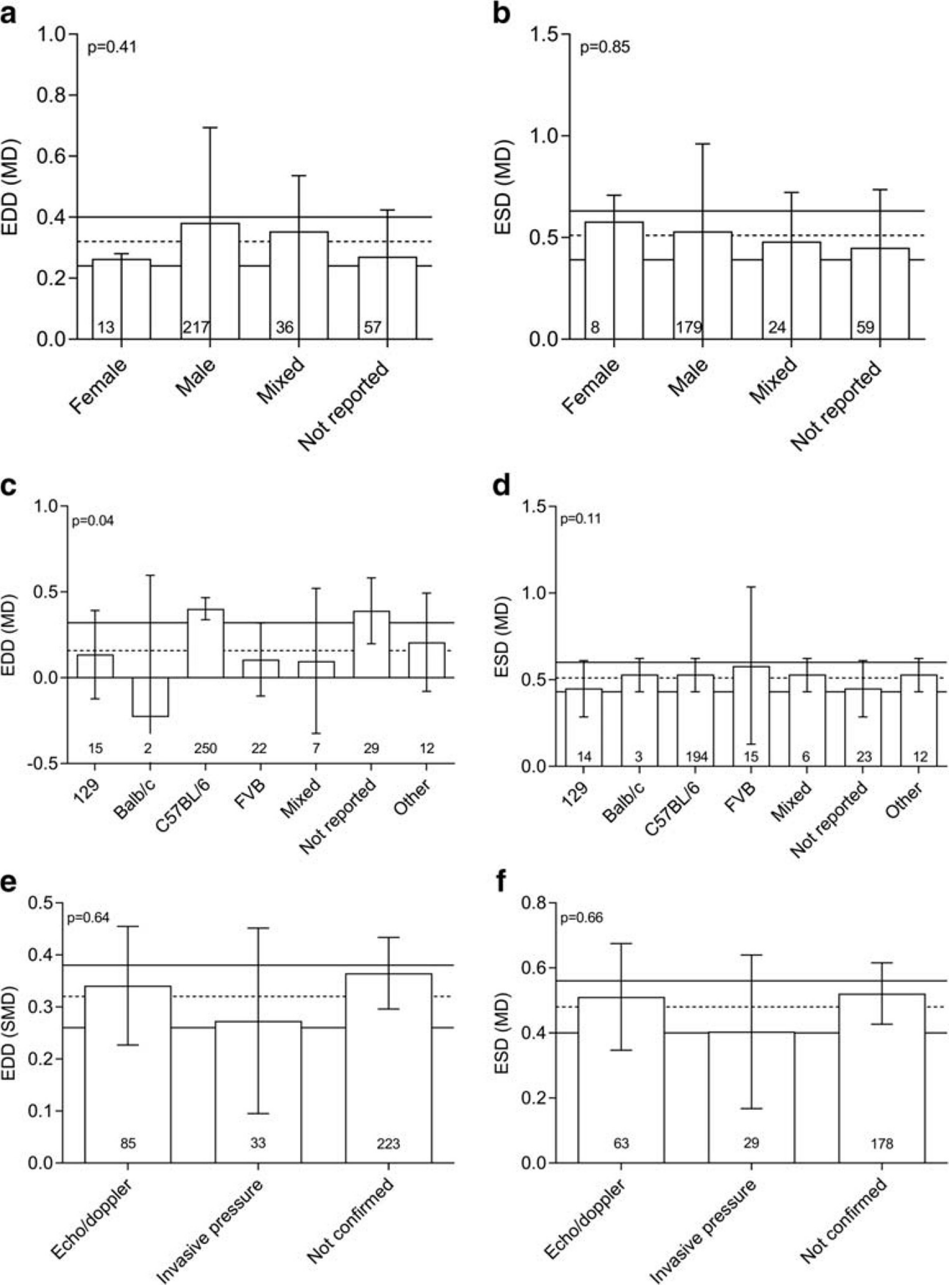
Influence of sex, mouse strain, and confirmation of pressure overload on end diastolic and end systolic diameters. Effect of sex (**a**), mouse strain (**b**), and confirmation of pressure overload on the outcome parameters EDD and ESD (MD). Horizontal dotted and solid lines represent pooled effect and its 95% confidence interval. In each bar, the number of comparisons contributing data is indicated

TAC surgery was effective in both sexes and did not differ depending on the sex of the animals (male versus female versus mixed versus not known) for any of the outcome measures (Fig. 6 a and b show the influence of sex on EDD and ESD, respectively). The strain of the mouse also did not account for a significant proportion of the heterogeneity in the overall analysis. The major differences are observed between C57Bl/6 and Balb/C mice; however, it must be noted that these outcomes may be biased as a consequence of the large proportion of studies that used C57BL/6, while only very limited numbers used Balb/c mice. In Fig. 6 c and d, the effect of mouse strain on EDD and ESD (MD) is shown. Also confirmation of proper placement of the TAC and thus cardiac pressure overload did not affect EDD and ESD (Fig. 6e, f).

### Influence of duration and diameter of constriction on TAC severity

**Fig. 7:**
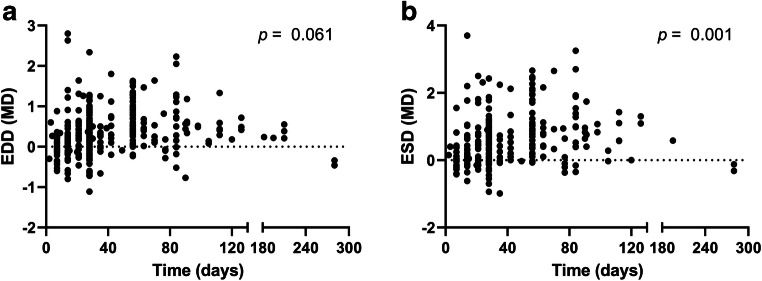
Correlation between duration of TAC and end diastolic and end systolic diameters. Effect of duration on TAC on EDD (**a**) and ESD (**b**) increase. Graph depicts mean difference in EDD and ESD for each individual comparison. *p* value derived from linear regression analysis

The duration of constriction varied from 3 to 280 days after TAC placement. Linear regression showed borderline significance for the effect of TAC duration on the primary outcome EDD (*p* = 0.061, Fig. 7a), while a stronger effect of constriction duration on the ESD was observed (*p* = 0.001, Fig. 7b). The constriction diameter of the needle used to apply the TAC varied from 17 to 30G. However, most data clustered around 27G. Linear regression did not show a significant relation between constriction diameter and EDD (*p* = 0.117) or ESD (*p* = 0.088, data not shown).

## Discussion

This systematic review and meta-analyses provide an overview of the commonly used animal model to study adverse cardiac remodeling: transverse aortic constriction (TAC). As expected, TAC significantly increased the EDD, EDV, ESD, and ESV indicative of adverse cardiac remodeling and heart failure.

### Animal characteristics

A clear preference for certain animal characteristics was seen. First, only rodents (mice and rats) were included in our study. During the screening phase, we noticed that in large animals (such as pigs and dogs), only a constriction of the ascending or descending aorta is used. Therefore, these animal species were not included in our review. Second, most of the articles used male animals, a very little proportion of studies was performed in female animals only. A troublesome 21% failed to report the sex of the animals. This sex bias, which is widespread in clinical and preclinical studies, is reason for concern. [12] Previously, a systematic review showed that the sex of the animals used was not reported in 20% of published cardiovascular animal research. Males were exclusively used in the majority (72%) of studies, whereas only 13% of articles reported the use of female animals. [13] These numbers are similar to our findings in for TAC model. In subgroup analysis, we were not able to show any effects of sex on the outcome parameters. Clinical studies document that female patients with similar degree of aortic stenosis more frequently demonstrate thick-walled chambers, corresponding with concentric left ventricular hypertrophy, compared with male patients. [14, 15] However, in preclinical studies, the distinguishing between concentric and eccentric hypertrophy is rarely made. Regarding mouse strain, a clear preference is seen for C57BL/6, used in 73% of the included mouse studies. A previous study showed that even the specific substrains of C57BL/6 differ in their (hypertrophic) response to TAC, indicating that genetic background might be of influence on the cardiac outcome after TAC. [16] However, in our subgroup analysis, no statistical significant proportion of between-study heterogeneity could be attributed to mouse strain for any outcome measure. However, this may be due to limited data available for certain strains or a lack of reporting on the specific C57BL/6 substrain used.

### Surgery characteristics

The standard surgery procedure in TAC models is to perform a partial thoracotomy for visualization of the aortic arch. This open-chest model leads to changes in respiratory physiology caused by the thoracotomy. To prevent this, a minimally invasive surgical technique through the 2nd intercostal space was developed, leading to a more rapid recovery of the mice. [17] Minimal invasive surgery was performed in 35% of the included studies. After surgery, 34% of studies mentioned confirmation of the TAC by either echo or invasive pressure measurements. This means that a relatively large proportion of studies may not have confirmed the constriction. Doppler echocardiography is a relatively easy and noninvasive technique to assess TAC severity without compromising future vascular access. [5] From our own experience, we know that sometimes a suture loosens, preventing adverse cardiac remodeling from happening. The low percentage of TAC confirmation might explain why a proportion of studies showed no or only very little adverse remodeling.

The majority of studies (71%) made use of 27G needles for TAC placement. No association between needle size and the outcome parameters was however observed. This is possibly due to clustering of data points around 27G since relatively large or small needles were less frequently used. Interestingly, only a marginal significant effect of TAC duration on one outcome parameter (EDD) was present. This was unexpected since a longer duration of TAC would generally lead to increased adverse remodeling and changes in all outcomes. Absence of association there might point towards inconsistency of the model causing large variability between animals and studies.

### Effect of TAC on the outcome parameters

When compared with control animals, TAC results in an increase in cardiac diameters and volumes (EDD, EDV, ESD, and ESV). Mice subjected to TAC exhibit variable severity of adverse remodeling. It was previously shown that independent of study characteristics, such as sex, strain, needle size, or time course, considerable variability exists within the model even when performed at the same laboratory. [5] Part of the animals show compensated LV hypertrophy, while only a subset of animals develop overt HF. Understanding this variability involved in the model is crucial to appropriate study design and interpretation, particularly when effects of conditional expression of genetic interventions or pharmacologic agents are studied. Variability within the model will affect the sample size required to detect the effect of treatment or genetic alterations. Of the included studies, 16–17% used ≤ 5 animals in the TAC group, and since only 7 out of 371 studies reported a power calculation, reproducibility of these results might be of concern [5]. The original Rockman technique [3] induces an identical flow area for each animal, regardless of its habitus, consequently causing a variable degree of aortic constriction. This leads to a greater pressure overload on the LV in heavier animals compared with smaller ones. Adjusting the constriction to the size of the mouse and the aorta can lead to more homogeneity of the results. [18] In our sub-analysis, we did not observe a significant influence of the average weight in each study. However, individual differences between mice per study are likely to exist.

### Study quality

Seventy-four percent of included papers mentioned a conflict of interest statement, of which most reported no conflicts of interest. Unfortunately, the other study quality indicators were very poorly reported. Only 23% reported blinding of the investigator during the study at any level, and only 10 studies reported a sample size calculation. Most articles did not report the number of animals used for each outcome measure. Adequate reporting of these methodological details is crucial to determine the risk of bias and to assess the quality of the evidence. [19] When the TAC model is used to study effects of treatment on adverse remodeling, underreporting of research methodology is often associated with an overestimation of effects. [20] As previously mentioned, we noticed that the number of animals in some studies was very low, and this might contribute to translational failure of preclinical studies. Since most of the studies did not include a power calculation, we cannot exclude the possible effect of under powering on our meta-analysis.

### Limitations of this review

Twenty-three studies were excluded because no animal numbers were reported. Furthermore, we had to exclude various articles because we could not confirm whether TAC was performed or another type of aortic banding. Both reasons indicate also low quality of methodological reporting and increase the risk of bias. However, because we excluded these papers, we cannot state this with certainty. In our search, we used for our control group both sham-operated animals as not-operated animals. This could have caused variation in our data, which we did not take into account. Data extraction was mainly performed by one reviewer, but because of the small between-reviewer deviations observed in the random sample, the effect is likely to be small. A full risk of bias assessment was not yet performed, and the very high amount of heterogeneity remains unexplained.

## Conclusion

The general TAC study is performed using a similar set-up as originally described by Rockman et al. [3], using male C57BL/6 mice of 10 weeks old with ketamine and xylazine as anesthetic, tightening the constriction around a 27G needle using a silk suture. Follow-up time is generally 28 days, and echocardiographic outcome parameters are assessed using 2D echocardiography. The murine TAC model is a very valuable but also variable animal model. For the future, especially when the TAC model is used to test pharmacological interventions, it is imperative that adequate numbers of mice are used, both sexes are studied, and the severity of constriction is confirmed after TAC or new operation techniques are applied to limit variability within the model.

.

## Electronic supplementary material

ESM 1 (PDF 259 kb)
