# Supplementary material for: The transverse aortic constriction heart failure animal model: a systematic review and meta-analysis
Source: Heart Fail Rev. 2020 Apr 25;26(6):1515–24. doi: 10.1007/s10741-020-09960-w (PMC8510918; doi:10.1007/s10741-020-09960-w)
Supplement: Supplementary file 1 — (PDF 259 kb) [file 10741_2020_9960_MOESM1_ESM.pdf]

Supplemental Material

SYSTEMATIC REVIEW PROTOCOL FOR ANIMAL INTERVENTION STUDIES

FORMAT BY SYRCLE (WWW.SYRCLE.NL)

VERSION 2.0 (DECEMBER 2014)

Item #	Section/Subsection/Item	Description	Check for approval
	A. General		
1.	Title of the review	Animal models of adverse cardiac remodeling after transverse aortic constriction: the influence of species, strain and sex.	
2.	Authors (names, affiliations, contributions)	J. de Haan, Experimental Cardiology, UMC Utrecht, Utrecht, The Netherlands<br>L. Bosch, Experimental Cardiology, UMC Utrecht, Utrecht, The Netherlands<br>K. Wever, SYRCLE, Radboudumc, Nijmegen, The Netherlands<br>G. Pasterkamp, Experimental Cardiology, UMC Utrecht, Utrecht, The Netherlands<br>H. el Azzouzi, Experimental Cardiology, Utrecht, The Netherlands<br>S. de Jager, Experimental Cardiology, UMC Utrecht, Utrecht, The Netherlands	
3.	Other contributors (names, affiliations, contributions)	None	
4.	Contact person + e-mail address	J. de Haan, j.j.dehaan-4@umcutrecht.nl	
5.	Funding sources/sponsors	ZonMw	
6.	Conflicts of interest	none	
7.	Date and location of protocol registration	6-3-2017<br>www.syrcle.nl	
8.	Registration number (if applicable)	NA	
9.	Stage of review at time of registration	Preliminary searches completed	
	B. Objectives		
	Background		

10.	What is already known about this disease/model/intervention? Why is it important to do this review?	There are various ways to study adverse cardiac remodeling after pressure-overload in animal models. These models resemble patients with aortic stenosis and pressure overload caused by hypertension. Transverse aortic constriction (TAC) is the most commonly used model. Different animal species (mainly mice and rats), strains and sexes are used. Currently it is unknown what the differences in severity of adverse remodeling and mortality after TAC are among species, strains and between sexes. We noticed in our own experiments that there are differences in the severity of cardiac remodelling and the mortality rate in response to pressure overload in strain and sex, but what those differences are exactly are not know. It is important to know what the differences are in response to pressure overload between strains and sex, so that a more grounded decision can be made to choose for a specific animal model, strain and sex.	
	Research question		
11.	Specify the disease/health problem of interest	Pressure-overloaded adverse cardiac remodelling after experimental transverse aortic constriction	
12.	Specify the population/species studied	Animals	
13.	Specify the intervention/exposure	Transverse aortic constriction (TAC)	
14.	Specify the control population	Animals without transverse aortic constriction	
15.	Specify the outcome measures	Adverse cardiac remodeling by end-systolic, end-diastolic volume (ESV & EDV), end-systolic, end-diastolic diameter (ESD&EDD), fractional shortening (FS) and ejection fraction (EF).	
16.	State your research question (based on items 11-15)	What is the effect of transverse aortic constriction on negative cardiac remodelling?<br><br>Which study characteristics, e.g. species, sex and strain, influence negative cardiac remodelling after TAC?	
	C. Methods		
	Search and study identification		
17.	Identify literature databases to search (*e.g.* Pubmed, Embase, Web of science)	X MEDLINE via PubMed □Web of Science<br>□SCOPUS X EMBASE<br>□Other, namely:<br>□Specific journal(s), namely:	
18.	Define electronic search strategies (*e.g.* use the step by step search guide[15] and animal search filters[20, 21])	When available, please add a supplementary file containing your search strategy: [insert file name]	

19.	Identify other sources for study identification	□Reference lists of included studies □Books<br>X Reference lists of relevant reviews<br>□Conference proceedings, namely:<br>□Contacting authors/ organisations, namely:<br>□Other, namely:	
20.	Define search strategy for these other sources	Reference list of relevant reviews will be screened on possible interesting titles. These papers will be screened with the same procedure as the references that came out of the initial search.	
	Study selection		
21.	Define screening phases (*e.g.* pre-screening based on title/abstract, full text screening, both)	1) screening for eligibility based on title/abstract<br>2) definitive inclusion or exclusion based on full text	
22.	Specify (a) the number of reviewers per screening phase and (b) how discrepancies will be resolved	a) 2 for each phase<br>b) Discrepancies will be resolved through discussion whenever possible. If consensus cannot be reached, a third reviewer will serve as arbiter	
	Define all inclusion and exclusion criteria based on:		
23.	Type of study (design)	Inclusion criteria: controlled studies with separate treatment arms<br>Exclusion criteria: No control group	
24.	Type of animals/population (*e.g.* age, gender, disease model)	Inclusion criteria: transverse aortic constriction (TAC) in all animal species, all different strains and sexes<br>Exclusion criteria: in vitro, ex vivo and clinical studies; animals with co-morbidities, genetically modified animals, animals undergoing co-intervention such as compound or solvent (except for PBS) administration; abdominal aortic constriction, Angiontensin II infusion, other ways of inducing hypertension /pressure overload.	
25.	Type of intervention (*e.g.* dosage, timing, frequency)	Inclusion criteria: TAC, all duration and all constriction diameters<br>Exclusion criteria: none	
26.	Outcome measures	Inclusion criteria: Cardiac function measured by echocardiography or MRI, cardiac hypertrophy, mortality<br>Exclusion criteria: no relevant outcomes reported	
27.	Language restrictions	Inclusion criteria: All languages<br>Exclusion criteria: none	
28.	Publication date restrictions	Inclusion criteria: all<br>Exclusion criteria: none	
29.	Other	Inclusion criteria: Full publication with original data<br>Exclusion criteria: conference abstract, short reports, letters to the editor, editorials.	
30.	Sort and prioritize your exclusion criteria per selection phase	Selection phase: Abstract/Title<br>1. not an original full publication (e.g. abstract, review)<br>2. not an in vivo animal study<br>3. no TAC model used	

		Selection phase: Full text<br>1. not an original full publication (e.g. abstract, review)<br>2. not an in vivo animal study<br>3. no TAC model used<br>4. no relevant outcome measures reported<br>5. unsuitable co-intervention applied<br>6. no suitable control group	
	Study characteristics to be extracted (for assessment of external validity, reporting quality)		
31.	Study ID (*e.g.* authors, year)	Authors, year, language	
32.	Study design characteristics (*e.g.* experimental groups, number of animals)	Sham/baseline, number of animals,	
33.	Animal model characteristics (*e.g.* species, gender, disease induction)	Strain, sex, age, weight, species	
34.	Intervention characteristics (*e.g.* intervention, timing, duration)	follow-up time, gauge needle/constriction diameter, TAC confirmation,	
35.	Outcome measures	Primary outcome<br>Cardiac function:<br>ESV or ESD<br>EDV or EDD<br><br>Secondary outcomes<br>EF or FS<br>Cardiac hypertrophy (heart weight/body weight, Heart weight/tibia length, ventricle weight/body weight, ventricle weight/tibia length)<br>Mortality	
36.	Other (*e.g.* drop-outs)	Number and reason of drop-outs per experimental group.	
	Assessment risk of bias (internal validity) or study quality		
37.	Specify (a) the number of reviewers assessing the risk of bias/study quality in each study and (b) how discrepancies will be resolved	a) 2<br>b) Discrepancies will be resolved through discussion whenever possible. If consensus cannot be reached, a third reviewer will serve as arbiter	
38.	Define criteria to assess (a) the internal validity of included studies (*e.g.* selection, performance, detection and attrition bias) and/or (b) other study quality measures (*e.g.* reporting quality, power)	□By use of SYRCLE's Risk of Bias tool[4]<br><br>X By use of SYRCLE's Risk of Bias tool, adapted as follows:<br><br>- Reporting of randomisation<br>- Reporting of blinding<br>- Reporting of sample size calculation<br>- Compliance with Animal welfare regulations<br><br>□By use of CAMARADES' study quality checklist, e.g [22]<br><br>□ By use of CAMARADES' study quality checklist, adapted as follows:<br><br>□Other criteria, namely:	
	Collection of outcome data		
39.	For each outcome measure, define the type	ESV: continuous, mm3	

	of data to be extracted (*e.g.* continuous/dichotomous, unit of measurement)	ESD: continuous, mm<br>EDV: continuous, mm3<br>EDD: continuous, mm<br>EF: continuous, %<br>FS: continuous, %<br>Mortality: incidence<br>Cardiac hypertrophy: heart weight/body weight ratio or heart weight/tibia length ratio	
40.	Methods for data extraction/retrieval (*e.g.* first extraction from graphs using a digital screen ruler, then contacting authors)	1. Numerical data mentioned in text<br>2. Extraction from graphs using a digital screen ruler	
41.	Specify (a) the number of reviewers extracting data and (b) how discrepancies will be resolved	a) 1, random check by second person. Digital ruler by 2 persons.<br>b) Discrepancies will be resolved through discussion whenever possible. If consensus cannot be reached, a third reviewer will serve as arbiter	
	Data analysis/synthesis		
42.	Specify (per outcome measure) how you are planning to combine/compare the data (*e.g.* descriptive summary, meta-analysis)	Descriptive summary, or meta-analysis when possible	
43.	Specify (per outcome measure) how it will be decided whether a meta-analysis will be performed	For all outcome measures:<br>Descriptive summary for outcomes reported in less than five articles.<br>Meta-analysis for outcomes reported in five or more articles	
	If a meta-analysis seems feasible/sensible, specify (for each outcome measure):		
44.	The effect measure to be used (*e.g.* mean difference, standardized mean difference, risk ratio, odds ratio)	ESV: standardized mean difference<br>EDV: standardized mean difference<br>ESD: standardized mean difference<br>EDD: standardized mean difference<br>FS: standardized mean difference<br>EF: standardized mean difference<br>Mortality: risk ratio,<br>Hypertrophy: standardized mean difference<br>N.B. if any of the SMD analyses contain data of only one species, the mean difference will be used.	
45.	The statistical model of analysis (*e.g.* random or fixed effects model)	Random effects model	
46.	The statistical methods to assess heterogeneity (*e.g.* I^2, Q)	I2 and/or R2	
47.	Which study characteristics will be examined as potential source of heterogeneity (subgroup analysis)	All species pooled: species, sex, blinding of outcome assessments, randomisation of allocation (pooling all species)<br>Corrected for or separated per species: constriction diameter(Gauge needle), age, strain, duration, and weight	
48.	Any sensitivity analyses you propose to perform	For mortality: Odds ratio instead or risk ratio<br>We aim to pool MRI and echo data, but we will do a sensitivity analyses to check whether the two different methods for cardiac function assessment matters.	

49.	Other details meta-analysis (*e.g.* correction for multiple testing, correction for multiple use of control group)	If applicable, we will perform a Holm-Bonferroni correction for testing multiple subgroups. If one or more subgroup analyses cannot be performed due to insufficient data, the p-value will be adjusted accordingly. Also correction for multiple use of control group will be performed by dividing the number of animals in the control group by the number of comparisons performed with this control group.	
50.	The method for assessment of publication bias	Produce funnel plots and visual analysis of these plots for outcome measures containing 20+ studies. For SMDs, we will use an n-based precision estimate to avoid distortion of the funnel plots. In addition, we aim to perform Egger's test for small study effects for outcome measures containing 20+ studies.	

Final approval by (names, affiliations):

Judith de Haan (UMCU)

Lena Bosch (UMCU)

Kim Wever (Radboud UMC)

Date: 6-3-2017

Supplemental tables

Supplemental table 1 *Pubmed search strategy*

(transverse aortic constriction[Title/Abstract]) OR (transverse aorta constriction[Title/Abstract]) OR (ascending aortic constriction[Title/Abstract]) OR (ascending aorta constriction[Title/Abstract]) OR (transthoracic aortic constriction[Title/Abstract]) OR (transthoracic aorta constriction[Title/Abstract]) OR (thoracic aortic constriction[Title/Abstract]) OR (thoracic aorta constriction[Title/Abstract]) OR (transverse aortic banding[Title/Abstract]) OR (transverse aorta banding[Title/Abstract]) OR (ascending aortic banding[Title/Abstract]) OR (ascending aorta banding[Title/Abstract]) OR (transthoracic aortic banding[Title/Abstract]) OR (transthoracic aorta banding[Title/Abstract]) OR (thoracic aortic banding[Title/Abstract]) OR (thoracic aorta banding[Title/Abstract]) OR pressure overload*[Title/Abstract]

Supplemental table 2 *Embase search strategy*

transverse:ti,ab AND aort*:ti,ab AND constriction:ti,ab OR (ascending:ti,ab AND aort*:ti,ab AND constriction:ti,ab) OR (transverse:ti,ab AND aort*:ti,ab AND banding:ti,ab) OR (ascending:ti,ab AND aort*:ti,ab AND banding:ti,ab) OR (transthoracic:ti,ab AND aort*:ti,ab AND constriction:ti,ab) OR
